# Synthesis of Spirocyclopropane-Containing 4*H*-Pyrazolo[1,5-*a*]indoles via Alkylative Dearomatization and Intramolecular *N*-Imination of an Indole–*O*-(Methylsulfonyl)oxime

**DOI:** 10.3390/molecules28176374

**Published:** 2023-08-31

**Authors:** Jiann-Jyh Huang, Hung-Chun Liao, Cheng-En Hsu, Yan-Ru Liu, Yi-Fu Chang, Shan-Yen Chou

**Affiliations:** 1 Institute of BioPharmaceutical Sciences, National Sun Yat-sen University, Kaohsiung 80424, Taiwan; 2 Department of Applied Chemistry, National Chiayi University, Chiayi City 600, Taiwan; 3 Development Center for Biotechnology, National Biotechnology Research Park, Taipei City 115, Taiwan

**Keywords:** 4*H*-pyrazolo[1,5-*a*]indole, pyrazoloindole, spirocyclopropane, alkylative dearomatization, *N*-imination, indole, *O*-(methylsulfonyl)oxime

## Abstract

In this paper, we report the synthesis of spirocyclopropane-containing 4*H*-pyrazolo[1,5-*a*]indoles **6a**–**e** via alkylative dearomatization and intramolecular *N*-imination of indole–*O*-(methylsulfonyl)oxime **11**. Starting materials tryptophol (**7**) and 2-bromocyclopetanone (**8**) were reacted in the presence of HBF_4_·OEt_2_, providing 1,2,3,5,6,11-hexahydrocyclopenta[2,3]oxepino[4,5-*b*]indole (**9**) in a 63% yield. Compound **9** was reacted with hydroxylamine hydrochloride to afford oxime **10** (65% yield), which was subsequently bis-methanesulfonated to form **11** in a 85% yield. Heating **11** with various alcohols in the presence of *N*,*N*-diisopropylethylamine (DIPEA) triggered the alkylative dearomatization and intramolecular *N*-imination, forming the spirocyclopropane and 4*H*-pyrazolo[1,5-*a*]indole structures in the targets **6a**–**e** with 67–84% yields.

## 1. Introduction

**Figure 1:**
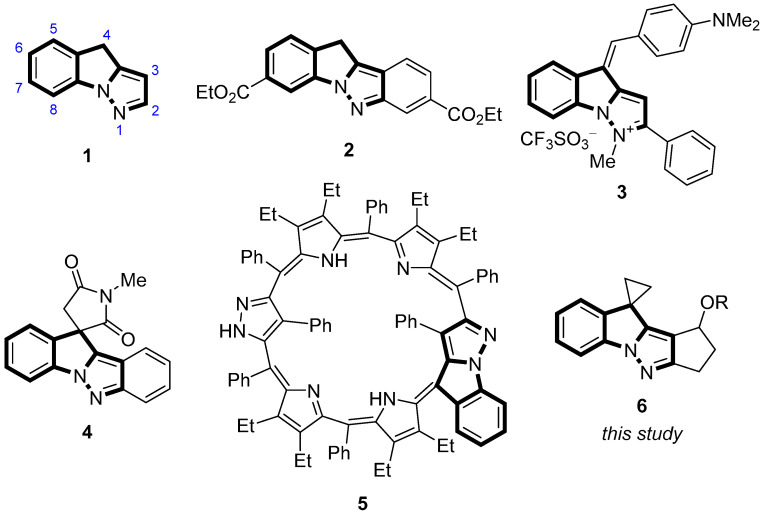
The structures of 4*H*-pyrazolo[1,5-*a*]indole (**1**), representative examples **2**–**5** containing **1**, and spirocyclopropane-containing 4*H*-pyrazolo[1,5-*a*]indole **6** reported in this study.

Pyrazolo[1,5-*a*]indole, a condensed type of pyrazoloindole, has three possible isomers. The 4*H*-Pyrazolo[1,5-*a*]indole (**1**, Figure 1) is the most stable isomer as the 1*H*-isomer is isoelectronic to azulene [1,2], and the 3*H*-isomer is readily converted to **1** [3,4]. As a result, pyrazoloindole **1** and its derivatives are more frequently reported. Representative examples using **1** as their scaffold or substructure are also 199shown in Figure 1, including diester **2** with a benzo-fused structure [5], salt **3** substituted with an exocyclic 4-methylene [6,7,8], benzo-fused **4** with a 4-spirosuccinimide [9], and Siamese-twin-type porphyrin **5 [10]**. Among them, compound **3** shows potent anti-cancer activity through inhibiting topoisomerase I [2], and compound **5** has an interesting folded structure [10].

**Scheme 1:**
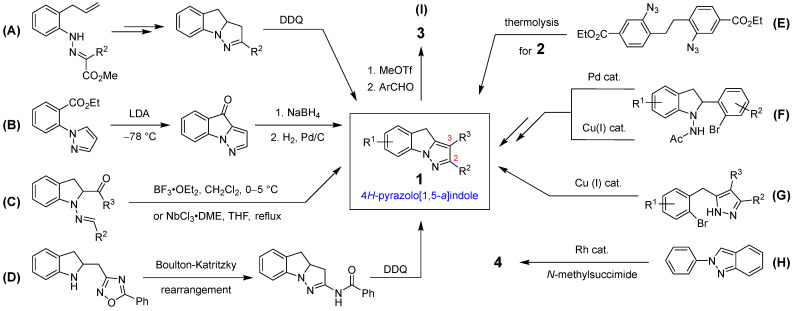
Reported methods for the synthesis of 4*H*-pyrazolo[1,5-*a*]indoles **1** [5,6,7,9,11,12,13,14,15,16,17,18,19].

Reported methods for the synthesis of 4*H*-pyrazolo[1,5-*a*]indole **1** are reviewed in Scheme 1. Katayama and his colleagues first reported the intramolecular cycloaddition of 2-allylphenylhydrazones to form the dihydropyrazoloindoles [11], followed by oxidation with DDQ [12] to synthesize 2-substituted **1** (Scheme 1A). Intramolecular cyclization of 1-(2′-carboethoxyphenyl)pyrazoles and subsequent reductive decarbonylation were also accomplished by Katayama to prepare 2-substituted **1** (Scheme 1B) [12]. The same group later reported the cyclization of hydrozone-containing indolines in the presence of Lewis acid (Scheme 1C) [13,14] and the use of Boulton–Katritzky rearrangement as the key step to synthesize 2-substituted **1** (Scheme 1D) [15]. Dougherty and his colleagues found the thermal decomposition of a diazide-generated benzo-fused **1** (Scheme 1E), which was the synthetic method for compound **2** shown in Figure 1 [5]. Pd-catalyzed [16] and Cu-catalyzed [17] aromatic amination of indolines were also reported by the Katayama group to prepare the benzo-fused derivatives of **1** (Scheme 1F). In another approach (Scheme 1G), Zhu and his colleagues applied Cu-catalyzed intramolecular *N*-arylation to pyrazoles to synthesize various 2- and/or 3-substituted derivatives of **1** [18], and the reaction could start from 1,3-diketones and hydrazine in tandem conditions [19]. Spiro **4** and its derivatives were prepared via the dehydrogenative annulation reaction of 2-arylindazoles with maleimides (Scheme 1H) [9]. As shown in Scheme 1I, salt **3** (Figure 1) was prepared by reacting the corresponding **1** with MeOTf and benzaldehyde [6,7]. Porphyrin **5** was prepared from the oxidation of its precedent Siamese-twin porphyrin [10].

**Scheme 2:**
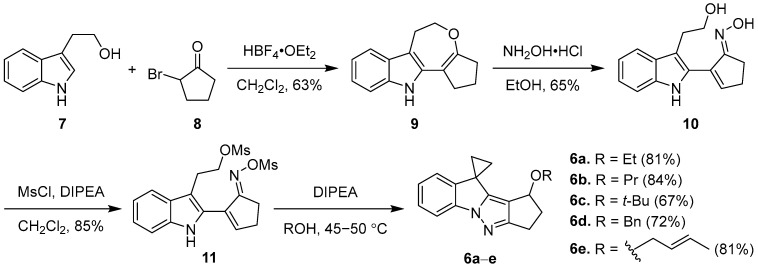
Synthesis of spirocyclopropane-containing 4*H*-pyrazolo[1,5-*a*]indoles **6a**–**e**.

We are interested in synthesizing novel heterocyclic compounds as they are often biologically active and possibly developed as pharmaceuticals [20]. In our discovering indolo[3,2-*c*]quinolinones as topoisomerase-I inhibitors [21], we found that spirocyclopropane-containing 4*H*-pyrazolo[1,5-*a*]indoles **6a**–**e** could be readily prepared from indole–*O*-(methylsulfonyl)oxime **11** through double cyclization reactions (Scheme 2). Substrate **11** was synthesized from tryptophol (**7**) and 2-bromocyclopentanone (**8**) through intermediates **9** and **10**. Compared with the synthetic methods from the literature (Scheme 1), our method has the advantage of using simple starting materials (indole and cycloalkanone) and avoiding the use of expensive or toxic metal catalysts to form **6a**–**e** having complex structures in good yields. In addition, **6a**–**e** are new compounds and have a characteristic cyclopropyl fragment that frequently appears in preclinical/clinical drug molecules [22], which might render them biologically active. Furthermore, the functional groups in **6a**–**e**, such as the alkoxy and spirocyclopropane, could further be modified to give derivatives with more diverse substituents.

Herein, we report the detailed reaction conditions for the transformations shown in Scheme 2. Based on the results, we also provided a tentative reaction mechanism to account for the cyclization of **11** to form **6a**–**e**.

## 2. Results and Discussion

We first found that the reaction of **7** with 2-chlorocyclopentanone (**12**) in the presence of pyridinium *p*-toluenesulfonate (PPTS, 20 mol %) in refluxing toluene for 2.0 h formed a trace amount of 1,2,3,5,6,11-hexahydrocyclopenta[2,3]oxepino[4,5-*b*]indole (**9**, entry 1, Table 1). As this reaction should take place through hemiacetal formation and Friedel–Crafts-like alkylation, the more reactive bromo substrate **8** was tried as the substrate. Nevertheless, the yield for **9** was not improved (entry 2). Using more acidic toluenesulfonic acid (TsOH) did not form the product regardless of using **8** or **12** (entries 3 and 4). Application of BF_3_·OEt_2_ (150 mol %) for the reaction with elongation of the reaction time to 3.0 h in CH_2_Cl_2_ at 0 °C gave the target **9** in 20% and 26% yields from substrates **12** and **8**, respectively (entries 5 and 6). When HBF_4_·OEt_2_ was used, the yields of **9** increased to 56% and 63% from the corresponding **12** and **8** (entries 7 and 8). Reduction of the amount of HBF_4_·OEt_2_ (75 mole %) decreased the yields of **9** (entries 9 and 10).

We then treated **9** with hydroxylamine hydrochloride (NH_2_OH·HCl) in EtOH and calculated the isolated yields of oxime **10** and enone **13** (Table 2). The reaction was found not to take place at room temperature (entry 1). At 55 °C, the reaction gave **10** and **13** in 63% and 27% yields, respectively (entry 2). Increasing the reaction temperature, the amount of NH_2_OH·HCl and the reaction time showed similar results (entries 3–5). As a result, enone **13** and its oxime **10** might be interconverted in equilibrium. The transformation comprised a benzylic oxidative reaction as a conjugated double bond was formed in **10** and **13**.

Mesylation of **10** with methanesulfonyl chloride (MsCl) and *N*,*N*-diisopropylethylamine (DIPEA) in CH_2_Cl_2_ afforded indole–*O*-(methylsulfonyl)oxime **11** (85% yield, Scheme 2), which was then reacted with various alcoholic solvents to afford the target spirocyclopropane-containing 4*H*-pyrazolo[1,5-*a*]indoles **6a**–**e**. Ethoxy analog **6a** was generated with an 81% yield. Propoxy analog **6b** had a better 84% yield. *Tert*-butoxy analog **6c** and benzyloxy analog **6d** showed slightly reduced yields (67% and 72%). The reaction of **11** with (*E*)-but-2-enol afforded the target **6e** an 81% yield with the retention of the *trans* configuration in the alkoxy group. When DIPEA for the reaction was replaced with secondary amines (e.g., Me_2_NH, Et_2_NH), a messy mixture was formed without the expected amino product. This might come from the reaction of the strong nucleophilic amines with the *O*-(methylsulfonyl)oxime or the cyclopropane moiety, which would result in the formation of multiple by-products.

The structures of the synthesized compounds **9**, **10**, **11**, **13**, and **6a**–**e** were fully characterized by spectroscopic methods (see Supplementary Materials). First, their molecular formulas were consistent with those suggested by high-resolution mass spectrometry. For the structure of **9**, five separated CH_2_ multipletes at 1.96–4.41 ppm in the ^1^H NMR spectrum suggested the presence of a cyclopentaoxepino moiety, in which the peaks at 4.28–4.41 ppm corresponded to the CH_2_ adjacent to the oxygen atom. A broad singlet at 7.68 ppm revealed an indolyl NH. The five most downfield peaks at 69.41 (OCH_2_), 34.12 (OCCH_2_), 30.32 (OC=CCH_2_), 27.74 (OCH_2_CH_2_), and 19.98 (CH_2_CH_2_CH_2_) ppm in the ^13^C NMR spectrum of **9** further supported the structure of a cyclopentaoxepino moiety.

For the structure of **10**, the presence of an oxime functionality was revealed by the stretching vibration bands at 3572 cm^–1^ (O–H), 1645 cm^–1^ (C=N), and 922 cm^–1^ (N–O) in the IR spectrum. Protons of the two CH_2_ in the cyclopentenone oxime moiety produced two multipletes at 2.72–2.77 and 2.85–2.89 ppm in the NMR spectrum, and the sole olefinic proton resided at 7.53–7.70 ppm. Protons of the two CH_2_ connected to the indole showed two tripletes centered at 3.23 and 3.96 ppm. In the ^13^C NMR spectrum of **10**, the β-carbon of the enone oxime showed a peak at 143.60 ppm. On the other hand, the spectra of **13** were similar to those of **10**, except that the carbonyl carbon showed a peak at 210.56 ppm. For **11**, similar ^1^H NMR patterns were observed. The two CH_3_ in the mesylate were located at 2.82 and 3.15 ppm in the ^1^H NMR spectrum and at 36.90 and 37.55 ppm in the ^13^C NMR spectrum.

For the final product **6a**, its ethoxy peaks were found at 1.21 ppm (triplet) and 3.51–3.40 ppm (multiplet) in the ^1^H NMR spectrum. Peaks at 1.63–1.87 ppm (multiplet) indicated the presence of a cyclopropane moiety, which was further confirmed by ^13^C NMR and DEPT spectrometry. The two methylene carbons were found at 17.81 and 16.79 ppm, and the quaternary carbon was found at 23.56 ppm. These results supported the structures of **6a** and could be referred to as the structures of **6b**–**e** bearing different alkoxy groups.

**Scheme 3:**
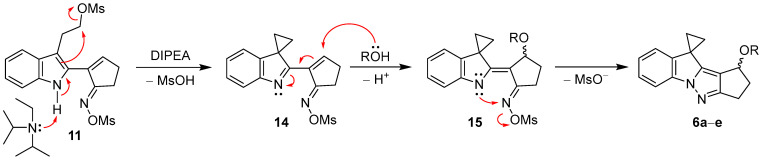
Tentative reaction mechanism for the conversion of **11** to **6a**–**e**.

For reaction mechanisms of the transformations shown in Scheme 2, we presume that the condensation of **7** and **8** to afford **9** took place by a hemiacetal formation and Friedel–Crafts-like alkylation, both promoted by the strong acid HBF_4_·OEt_2_ (step 1). The enol-ether-containing seven-membered ring in **9** should be opened by highly nucleophilic hydroxylamine followed by benzylic oxidation to form **10** (step 2). This step might be a novel tandem oximation/oxidation reaction that can be further investigated. Compound **10** was then converted to bis-methanesulfonated **11** by reacting with MsCl (step 3). The intriguing cyclization of **11** to **6a**–**e** (step 4) was accounted for in a tentative reaction mechanism presented in Scheme 3. DIPEA first deprotonated **11,** and the formed amide anion pushed the conjugated π electrons to expel the methanesulfonate anion (MsO^–^), forming the spirocyclopropane structure in **14**. This alkylative dearomatization reaction (**11** → **14**) might take place first as similar reactions were reported to proceed fast [23,24]. Intermediate **14**, having a cross-conjugation system, was attacked by alcohols to form anion **15** with a more stable N-anion, C=C, and C=N conjugation. Finally, the *N*-imination reaction occurred in **15** when the amide anion attacked the nitrogen atom of the oxime sulfonate ester to form the pyrazole moiety in the final products **6a**–**e**. The mechanism of this N–N formation was supported by the similar reactions reported by Stambuli and his colleagues for the synthesis of indazoles [25,26] and should be thermodynamically favored because of the formation of aromatic structure.

Oxime and its *O*-substituted derivative (i.e., the oxime sulfonate in **11**, Scheme 2 and Scheme 3) can be used as electron-deficient *N*-imination or amination agents [27]. In addition to the transformation of **11** to **6a**–**e** (Scheme 3) and the synthesis of indazole previously mentioned [25,26], **an** intramolecular reaction of *O*-(arylsulfonyl)oximes with a nearby amino group was applied to the synthesis of 1,2,3-triazoles [28]. Oximes activated by *N*,*N*’-dicyclohexylcarbodiimide were also used to synthesize pyrrolidines [29]. The intermolecular reaction of *O*-(arylsulfonyl)oximes with arylamines forms the corresponding arylhydrazones [30,31]. Arylamines, alkylamines, and sulfonamides can couple with 2-bromoaryl oxime acetates catalyzed by Cu(I) to afford various 1*H*-indazoles [32]. Therefore, the transformation of **11** to **6a**–**e** reported herein was a new application of *N*-imination with activated oximes for the synthesis of 4*H*-pyrazolo[1,5-*a*]indoles.

## 3. Materials and Methods

### 3.1. General Procedure

Reagents and starting materials were used as purchased without further purification. Purification by column chromatography was conducted using Merck Reagents Silica Gel 60 (particle size of 0.063–0.200 mm, 70–230 mesh ASTM). The melting point was recorded on a STUART SMP3 apparatus. Proton (300 MHz) and carbon-13 (75 MHz) NMR spectra were recorded on a Varian Mercury-300 spectrometer using CDCl_3_ as the solvent. Multiplicities are abbreviated as follows: s, singlet; d, doublet; t, triplet; q, quartet; m, multiplet; *J*, coupling constant (hertz). Infrared (IR) spectra were measured on a PerkinElmer ONE FT-IR spectrometer with an ATR accessory. High-resolution mass spectra were obtained on an LTQ Orbitrap XL mass spectrometer (Thermo Fisher Scientific).

### 3.2. Synthesis of Compounds ***9–11***

#### 3.2.1. 1,2,3,5,6,11-Hexahydrocyclopenta[2,3]oxepino[4,5-b]indole (**9**)

A solution of tryptophol (**7**, 5.50 g, 34.1 mmol) and freshly prepared 2-bromocyclopentanone (**8**, 7.11 g, 43.6 mmol) [33] in anhydrous CH_2_Cl_2_ (90 mL) was added with HBF_4_·OEt_2_ (11.04 g, 68.2 mmol) in a period of 5.0 min at 0 °C under N_2_. The reaction mixture was stirred at 0 °C for 10 h. The solution was diluted with CH_2_Cl_2_ (90 mL), quenched with icy water (100 mL), neutralized with saturated aqueous K_2_CO_3_, and filtered through Celite. The filtrate was washed with water, dried over MgSO_4_, and concentrated under reduced pressure. The residue was purified by column chromatography on silica gel using a mixture of EtOAc and hexanes (1:5) as the eluent to give the target **9** (4.83 g, 21.4 mmol) in 63% yield: brown oil; ^1^H NMR δ 7.68 (brs, 1 H), 7.47–7.40 (m, 1 H), 7.33–7.27 (m, 1 H), 7.13–7.06 (m, 1 H), 4.41–4.28 (m, 2 H), 3.21 (t, *J* = 4.4 Hz, 2 H), 2.82–2.71 (m, 2 H), 2.66–2.55 (m, 2 H), and 2.10–1.96 (m, 2 H); ^13^C NMR δ 157.70, 135.05, 132.13, 128.50, 120.84, 119.52, 117.10, 110.66, 110.48, 103.36, 69.41, 34.12, 30.32, 27.74, 19.98; IR 3443, 3386, 2961, 2924, 2889, 1629, 1460, 1327, 1274, 1098, and 731 cm^–1^; HRMS Calcd for [C_15_H_15_NO + H^+^]: 226.1226; Found: 226.1224; new compound.

#### 3.2.2. (E)-2-[3-(2-Hydroxyethyl)-1H-indol-2-yl]cyclopent-2-en-1-one Oxime (**10**)

A solution of **9** (2.70 g, 12.0 mmol) and hydroxylamine hydrochloride (1.80 g, 25.9 mmol) in 95% EtOH (100 mL) was heated under reflux for 15 h under N_2_. The solution was concentrated, re-dissolved in CH_2_Cl_2_ (20 mL), washed with 5% aqueous Na_2_CO_3_ (10 mL) and water (10 mL), and concentrated under reduced pressure. The residue was purified by column chromatography on silica gel using a mixture of EtOAc and hexanes (1:2) as the eluent to give **10** (2.00 g, 7.80 mmol) as white solids in 65% yield: mp 140.5–141.5 °C; ^1^H NMR δ 9.97 (brs, 1 H), 7.70–7.53 (m, 1 H), 7.36 (dt, *J* = 8.1, 1.0 Hz, 1 H), 7.23–7.07 (m, 3 H), 3.96 (t, *J* = 6.6 Hz, 2 H), 3.23 (t, *J* = 6.6 Hz, 2 H), 2.89–2.85 (m, 2 H), and 2.77–2.72 (m, 2 H); ^13^C NMR δ 168.37, 143.60, 135.05, 130.97, 128.60, 128.41, 122.50, 119.45, 118.61, 111.21, 110.40, 62.47, 29.97, 28.45, and 25.25; IR 3572, 3341, 3077, 2873, 1645, 1453, 1434, 1304, 1004, 922, and 740 cm^–1^; HRMS calculated for [C_15_H_16_N_2_O_2_ + H^+^]: 257.1285; Found: 257.1283; new compound.

#### 3.2.3. 2-[3-(2-Hydroxyethyl)-1H-indol-2-yl]cyclopent-2-en-1-one (**13**)

Compound **13** was purified by column chromatography from the reaction mixture to prepare **10**; white solids; mp 141.0–141.5 °C; ^1^H NMR δ 10.32 (brs, 1 H), 8.19 (t, *J* = 3.2 Hz, 1 H), 7.64–7.58 (m, 1 H), 7.41 (dt, *J* = 8.1, 1.1 Hz, 1 H), 7.22 (ddd, *J* = 8.1, 7.0, 1.1 Hz, 1 H), 7.12 (ddd, *J* = 8.0, 7.0, 1.1 Hz, 1 H), 3.95 (t, *J* = 6.5 Hz, 2 H), 3.23 (t, *J* = 6.5 Hz, 2 H), 2.88–2.79 (m, 2 H), and 2.65–2.57 (m, 2 H); ^13^C NMR δ 210.56, 158.05, 135.34, 134.59, 128.04, 127.52, 123.16, 119.82, 118.79, 111.76, 111.13, 62.79, 35.00, 28.68, and 27.41; IR 3401, 3093, 3080, 2840, 1742, 1622, 1354, 1268, 1125, and 1118 cm^–1^; HRMS calculated for [C_15_H_15_NO_2_ + H^+^]: 242.1176; Found: 242.1172; new compound.

#### 3.2.4. (E)-2-[2-(5-{[(Methylsulfonyl)oxy]imino}cyclopent-1-en-1-yl)-1H-indol-3-yl]ethyl Methanesulfonate (**11**)

An anhydrous CH_2_Cl_2_ solution (240 mL) of **10** (6.31 g, 24.6 mmol) and DIPEA (7.72 g, 59.7 mmol) in an ice bath was added with methanesulfonyl chloride (6.25 g, 54.6 mmol). The reaction mixture was stirred in an ice bath under N_2_ for 12 h. The solution was quenched with water (100 mL) and extracted with CH_2_Cl_2_ (50 mL × 2). The organic layer was washed with 1.0 N HCl (80 mL), water (80 mL), 10% NaHCO_3_ (80 mL), and brine. The solution was dried over anhydrous MgSO_4_, filtered, and concentrated under reduced pressure. The residue was purified by column chromatography on silica gel using a mixture of EtOAc and hexanes (1:2) as the eluent to give **11** (8.63 g, 20.9 mmol) as a brown oil in 85% yield: ^1^H NMR δ 9.44 (brs, 1 H), 7.57 (d, *J* = 7.8 Hz, 1 H), 7.39 (d, *J* = 7.8 Hz, 1 H), 7.28–7.22 (m, 2 H), 7.15–7.13 (m, 1 H), 4.42 (t, *J* = 7.5 Hz, 2 H), 3.36 (t, *J* = 7.5 Hz, 2 H), 3.15 (s, 3 H), 3.00–2.96 (m, 2 H), 2.82 (s, 3 H), and 2.80–2.78 (m, 2 H); ^13^C NMR δ 175.83, 150.43, 135.40, 130.04, 127.87, 127.11, 123.49, 120.35, 118.53, 111.80, 109.31, 69.20, 37.55, 36.90, 30.59, 27.75, and 25.67; IR 3491, 3096, 3085, 1668, 1303, 1299, 1286, 1138, 1130, and 1027; HRMS calculated for [C_17_H_20_N_2_O_6_S_2_ + H^+^]: 413.0836; Found: 413.0833; new compound.

### 3.3. Synthesis of 1-Alkoxy-2,3-dihydro-1H-spiro[cyclopenta[3,4]pyrazolo[1,5-a]indole-10,1′-cyclopropanes] ***6a–e***

#### 3.3.1. Standard Procedure

A reaction mixture of **11** (~200 mg, 1.0 equiv) and DIPEA (5.0 equiv) in an anhydrous alcoholic solvent (12 mL) was heated at 45–50 °C for 2.0 h. The solution was concentrated under reduced pressure, re-dissolved in CH_2_Cl_2_, dried over anhydrous MgSO_4_, filtered, and concentrated under reduced pressure. The residue was purified by column chromatography on silica gel using a mixture of EtOAc and hexanes (1:6) as the eluent to give **6a**–**e** as liquids in 67–84% yields.

#### 3.3.2. 1-Ethoxy-2,3-dihydro-1H-spiro[cyclopenta[3,4]pyrazolo[1,5-a]indole-10,1′-cyclopropane] (**6a**)

Using EtOH as the solvent; 81% yield; yellow liquid; ^1^H NMR δ 7.59 (d, *J* = 7.4 Hz, 1 H), 7.35–7.30 (m, 1 H), 7.16–7.09 (m, 1 H), 6.96 (d, *J* = 7.4 Hz, 1 H), 4.92–4.80 (m, 1 H), 3.54–3.42 (m, 2 H), 3.07–2.97 (m, 1 H), 2.81–2.64 (m, 2 H), 2.47–2.34 (m, 1 H), 1.91–1.71 (m, 4 H), and 1.22 (t, *J* = 7.0 Hz, 3 H); ^13^C NMR δ 166.06, 145.69, 140.50, 138.48, 127.14, 123.83, 119.46, 115.57, 110.12, 75.17, 63.37, 37.32, 23.81, 23.56, 17.81, 16.79, and 15.80; IR 3097, 3007, 2884, 1609, 1468, 1409, 1310, 1301, 1108, and 778 cm^–1^; HRMS calculated for [C_17_H_18_N_2_O + H^+^]: 267.1492; Found: 267.1488; new compound.

#### 3.3.3. 1-Propoxy-2,3-dihydro-1H-spiro[cyclopenta[3,4]pyrazolo[1,5-a]indole-10,1′-cyclopropane] (**6b**)

Using PrOH as the solvent;: 84% yield; brown liquid; ^1^H NMR δ 7.61–7.57 (m, 1 H), 7.35–7.30 (m, 1 H), 7.17–7.10 (m, 1 H), 6.98–6.94 (m, 1 H), 4.86 (dd, *J* = 6.6, 3.2 Hz, 1 H), 3.48–3.40 (m, 1 H), 3.37–3.29 (m, 1 H), 3.07–2.98 (m, 1 H), 2.82–2.67 (m, 2 H), 2.44–2.34 (m, 1 H), 1.85–1.64 (m, 6 H), and 0.96 (t, *J* = 7.3 Hz, 3 H); ^13^C NMR δ 165.94, 145.64, 140.45, 138.44, 127.07, 123.77, 119.42, 115.51, 110.05, 75.31, 69.83, 37.16, 23.79, 23.47, 23.37, 17.86, 16.67, and 10.91; IR 3098, 3041, 3006, 2885, 1468, 1460, 1375, 1310, 1302, and 1109, cm^–1^; HRMS calculated for [C_18_H_20_N_2_O + H^+^]: 281.1648; Found: 281.1654; new compound.

#### 3.3.4. 1-(Tert-butoxy)-2,3-dihydro-1H-spiro[cyclopenta[3,4]pyrazolo[1,5-a]indole-10,1′-cyclopropane] (**6c**)

Using *t*-BuOH and THF (3:1) as the solvent; 67% yield; brown liquid; ^1^H NMR δ 7.54 (d, *J* = 7.8 Hz, 1 H), 7.31–7.24 (m, 1 H), 7.11–7.06 (m, 1 H), 6.92 (d, *J* = 7.8 Hz, 1 H), 5.04 (t, *J* = 2.1 Hz, 1 H), 2.98–2.94 (m, 1 H), 2.77–2.70 (m, 2 H), 2.26 (m, 1 H), 1.94–1.91 (m, 1 H), 1.72–1.64 (m, 3 H), and 1.26 (s, 9 H); ^13^C NMR δ 164.77, 145.29, 140.60, 138.51, 127.19, 123.72, 119.54, 117.28, 110.09, 73.87, 68.76, 41.47, 31.42, 28.95, 24.31, 17.72, and 17.18; IR 3103, 3047, 3044, 3000, 1608, 1462, 1408, 1308, 1205, and 1077 cm^–1^; HRMS calculated for [C_19_H_22_N_2_O + H^+^]: 295.1805; Found: 295.1804; new compound.

#### 3.3.5. 1-(Benzyloxy)-2,3-dihydro-1H-spiro[cyclopenta[3,4]pyrazolo[1,5-a]indole-10,1′-cyclopropane] (**6d**)

Using BnOH and THF (1:1) as the solvent; 72% yield; yellow liquid; ^1^H NMR δ 7.60 (d, *J* = 7.5 Hz, 1 H), 7.40–7.31 (m, 6 H), 7.14 (t, *J* = 7.5 Hz, 1 H), 6.95 (d, *J* = 7.5 Hz, 1 H), 4.99 (dd, *J* = 6.5, 3.2 Hz, 1 H), 4.55 (d, *J* = 11.6 Hz, 1 H), 4.47 (d, *J* = 11.6 Hz, 1 H), 3.10–3.01 (m, 1 H), 2.82–2.68 (m, 2 H), 2.55–2.41 (m, 1 H), 1.92–1.85 (m, 1 H), and 1.78–1.68 (m, 3 H); ^13^C NMR δ 166.08, 145.78, 140.45, 138.62, 138.44, 128.59, 127.87, 127.60, 127.05, 123.89, 119.46, 110.20, 74.76, 70.05, 37.07, 23.82, 23.58, 17.95, and 16.79; IR 3087, 3072, 2850, 1678, 1608, 1470, 1309, 1085, 1407, and 1370 cm^–1^; HRMS calculated for [C_22_H_20_N_2_O + H^+^]: 329.1648; Found: 329.1655; new compound.

#### 3.3.6. (E)-1-(But-2-en-1-yloxy)-2,3-dihydro-1H-spiro[cyclopenta[3,4]pyrazolo[1,5-a]indole-10,1′-cyclopropane] (**6e**)

Using (*E*)-but-2-en-1-ol as the solvent; 81% yield; yellow liquid; ^1^H NMR δ 7.53 (d, *J* = 7.5 Hz, 1 H), 7.29–7.22 (m, 1 H), 7.09–7.06 (m, 1 H), 6.90 (d, *J* = 7.5 Hz, 1 H), 5.69–5.63 (m, 1 H), 5.58–5.50 (m, 1 H), 4.85–4.82 (m, 1 H), 3.86–3.82 (m, 2 H), 3.00–2.93 (m, 1 H), 2.75–2.57 (m, 2 H), 2.40–2.32 (m, 1 H), and 1.86–1.63 (m, 7 H); ^13^C NMR δ 166.16, 145.63, 140.47, 138.47, 129.43, 127.87, 127.13, 123.81, 119.43, 115.43, 110.12, 74.57, 68.76, 37.32, 23.76, 23.55, 17.95, 17.85, and 16.79; IR 2994, 2769, 2759, 1611, 1478, 1471, 1367, 1306, 1133, and 1081 cm^–1^; HRMS calculated for [C_19_H_20_N_2_O + H^+^]: 293.1648; Found: 293.1647; new compound.

## 4. Conclusions

We report a synthetic method for the preparation of spirocyclopropane-containing 4*H*-pyrazolo[1,5-*a*]indoles **6a**–**e** from indole–*O*-(methylsulfonyl)oxime **11**. Double cyclizations involving alkylative dearomatization and intramolecular *N*-imination were proposed as the reaction mechanism for the transformation. Currently, we are investigating intramolecular *N*-imination on simple indole substrates and the topoisomerase-I inhibitory activity of **6a**–**e**. The results will be reported in due course.

## Figures and Tables

**Table 1:**
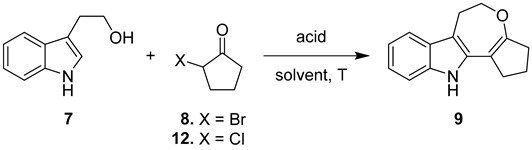
Optimization of the reaction conditions for the synthesis of **9** *^a^*.

Entry	X	Acid (mol %)	Solvent	T (°C) *^b^*	Time (h)	Yield (%) *^c^*
1	Cl	PPTS (20) *^d^*	toluene	reflux	2.0	Trace *^e^*
2	Br	PPTS (20) *^d^*	toluene	reflux	2.0	Trace *^e^*
3	Cl	TsOH (20)	toluene	reflux	2.0	0 *^f^*
4	Br	TsOH (20)	toluene	reflux	2.0	0 *^f^*
5	Cl	BF_3_·OEt_2_ (150)	CH_2_Cl_2_	0	3.0	20
6	Br	BF_3_·OEt_2_ (150)	CH_2_Cl_2_	0	3.0	26
7	Cl	HBF_4_·OEt_2_ (150)	CH_2_Cl_2_	0	10	56
8	Br	HBF_4_·OEt_2_ (150)	CH_2_Cl_2_	0	10	63
9	Cl	HBF_4_·OEt_2_ (75)	CH_2_Cl_2_	0	10	25
10	Br	HBF_4_·OEt_2_ (75)	CH_2_Cl_2_	0	10	30

*^a^* The reaction was carried out using **7** (~200 mg, 1.0 equiv), **8,** or **12** (1.2 equiv), and acids in 4.0 mL of solvents. *^b^* Bath temperature. *^c^* Isolated yield. *^d^* PPTS, pyridinium *p*-toluenesulfonate. *^e^* The [M + 1]^+^ peak of **9** was detected in ESI-MS. *^f^* The peaks related to **9** were not observed in ESI-MS.

**Table 2:**
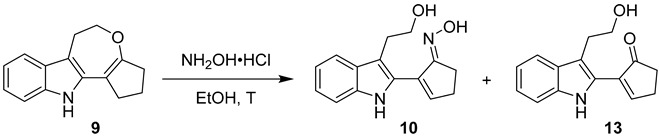
Optimization of the reaction conditions for oxime **10** *^a^*.

Entry	Equivalent of NH_2_OH·HCl	T (°C) *^b^*	Time (h)	Yield (%) *^c^*	
				10	13
1	2.2	r.t.	15	0 *^d^*	0 *^d^*
2	2.2	55	15	63	27
3	2.2	reflux	15	65	25
4	3.2	reflux	30	62	20
5	3.2	reflux	45	63	18

*^a^* The reaction was carried out using **9** (~200 mg, 1.0 equiv) with NH_2_OH·HCl in 10 mL of 95% EtOH. *^b^* Bath temperature. *^c^* I solated yield. *^d^* No reaction with the recovery of starting material.

## Data Availability

Not applicable.

## Supplementary Materials

The following supporting information can be downloaded at https://www.mdpi.com/article/10.3390/molecules28176374/s1, ^1^H and ^13^C NMR spectra of compounds **9**, **10**, **11**, and **6a**–**e**.

## Sample Availability

Samples of the compounds are available from the authors.
