# Supplementary material for: Synthesis of Spirocyclopropane-Containing 4H-Pyrazolo[1,5-a]indoles via Alkylative Dearomatization and Intramolecular N-Imination of an Indole–O-(Methylsulfonyl)oxime
Source: Molecules. 2023 Aug 31;28(17):6374. doi: 10.3390/molecules28176374 (PMC10490174; doi:10.3390/molecules28176374)

^{1}H and ^{13}C NMR Spectra of the Synthesized Compounds

9, 10, 13, 11, and 6a–e

Instrumental section

Proton (300 MHz) and carbon-13 (75 MHz) NMR spectra were recorded on a Varian Mercury-300 spectrometer at room temperature using $CDCl_3$ as the solvent. Proton chemical shifts are referenced to the residual $CHCl_3$ (δ 7.26 ppm) in $CDCl_3$. Carbon-13 chemical shifts are referenced to the center of the $CDCl_3$ triplet (δ 77.0 ppm). Multiplicities are abbreviated as follows: s, singlet; d, doublet; t, triplet; q, quartet; m, multiplet; *J*, coupling constant (hertz).

1,2,3,5,6,11-Hexahydrocyclopenta[2,3]oxepino[4,5-*b*]indole (9)

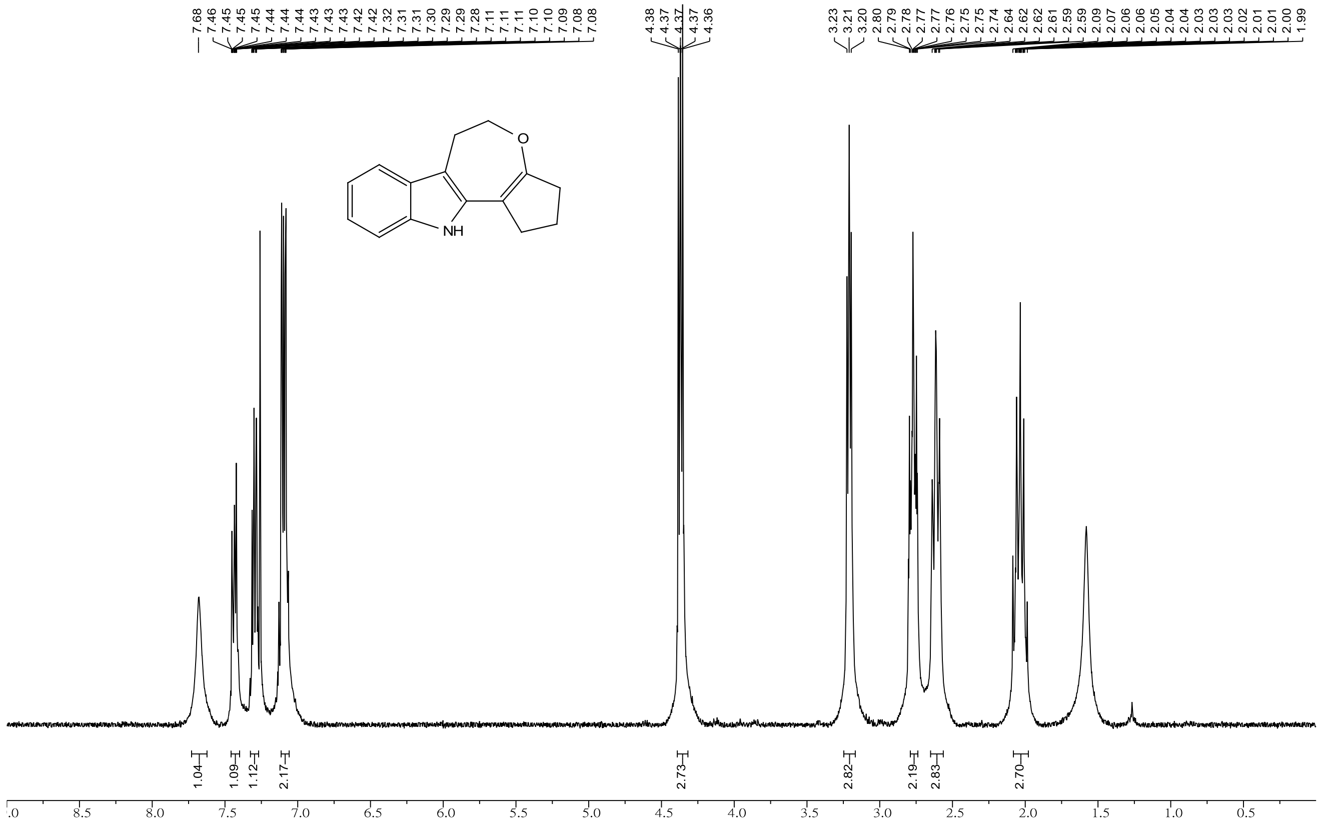

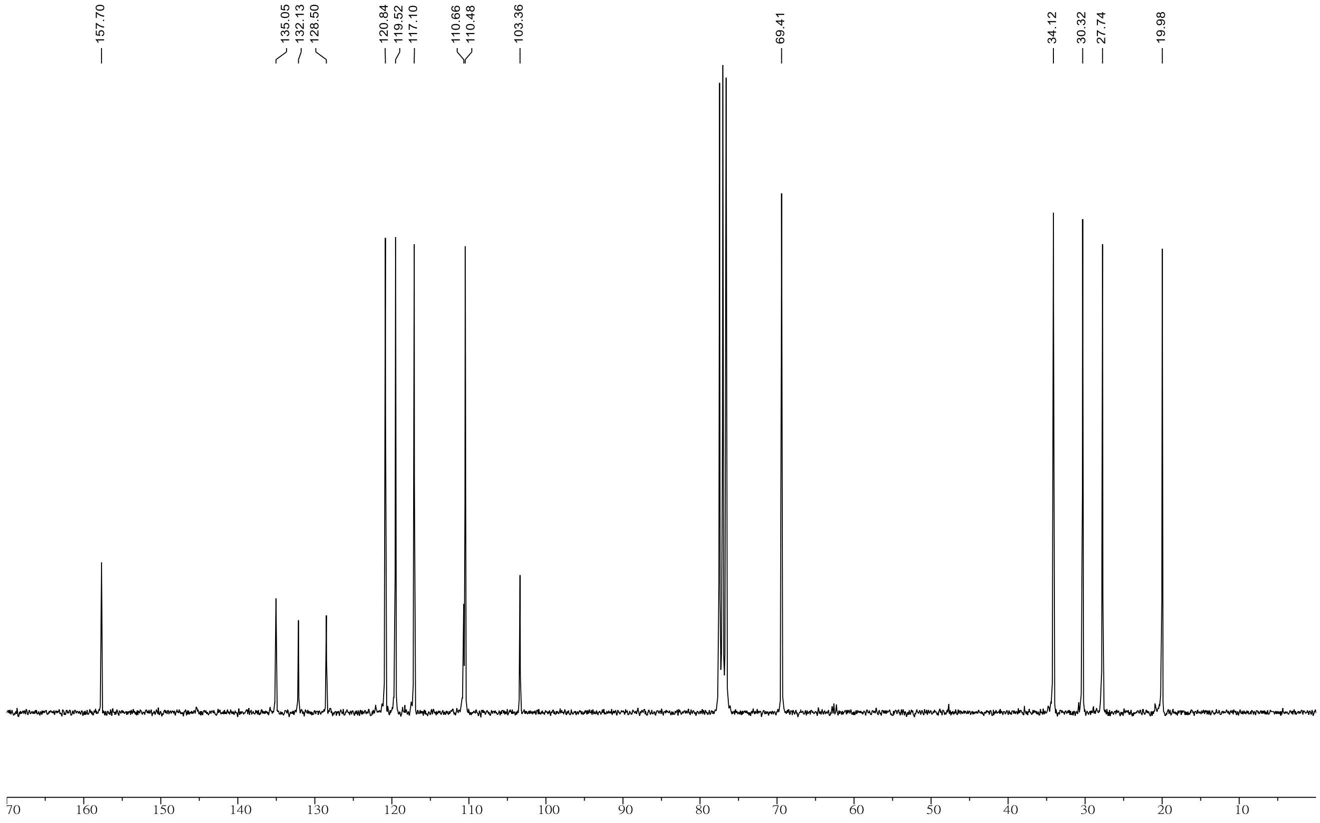

(*E*)-2-[3-(2-Hydroxyethyl)-1*H*-indol-2-yl]cyclopent-2-en-1-one Oxime (10)

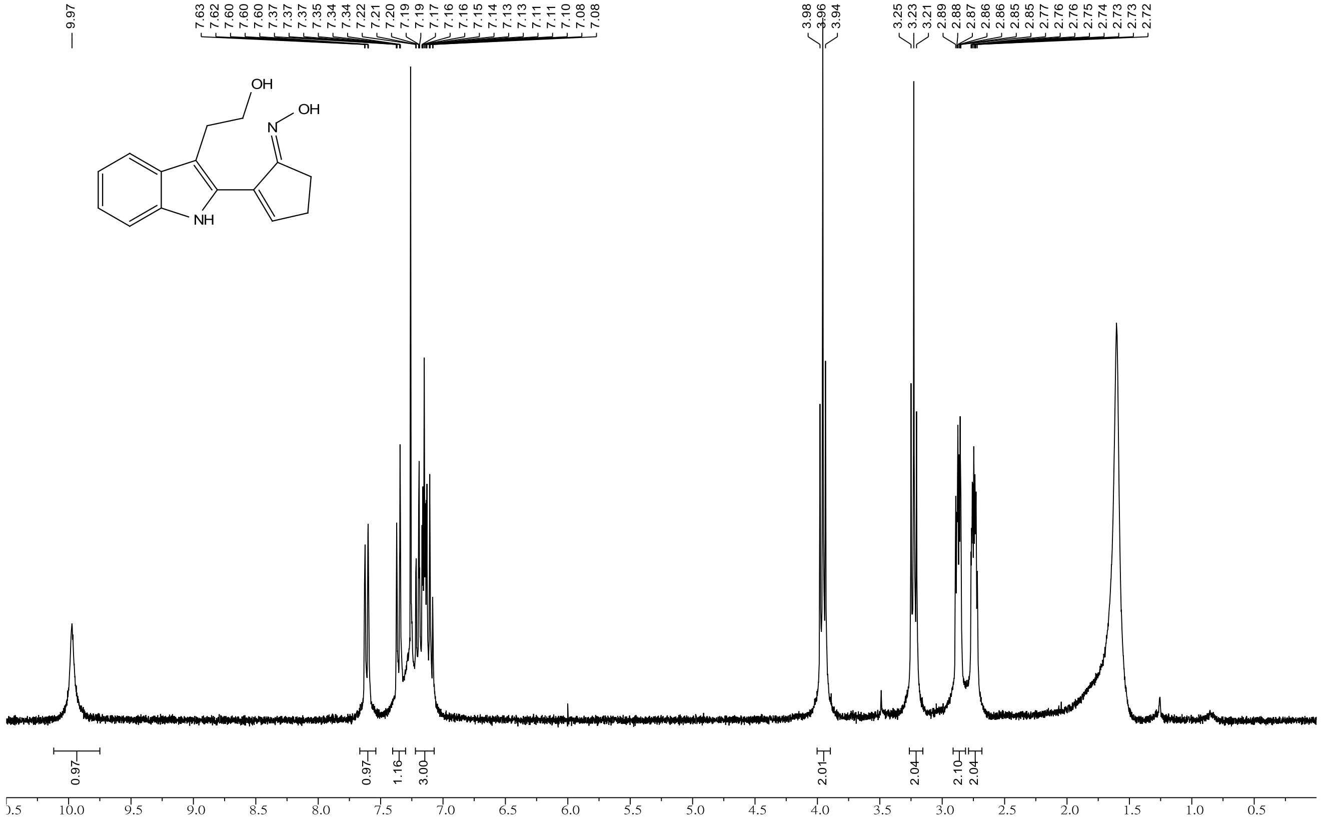

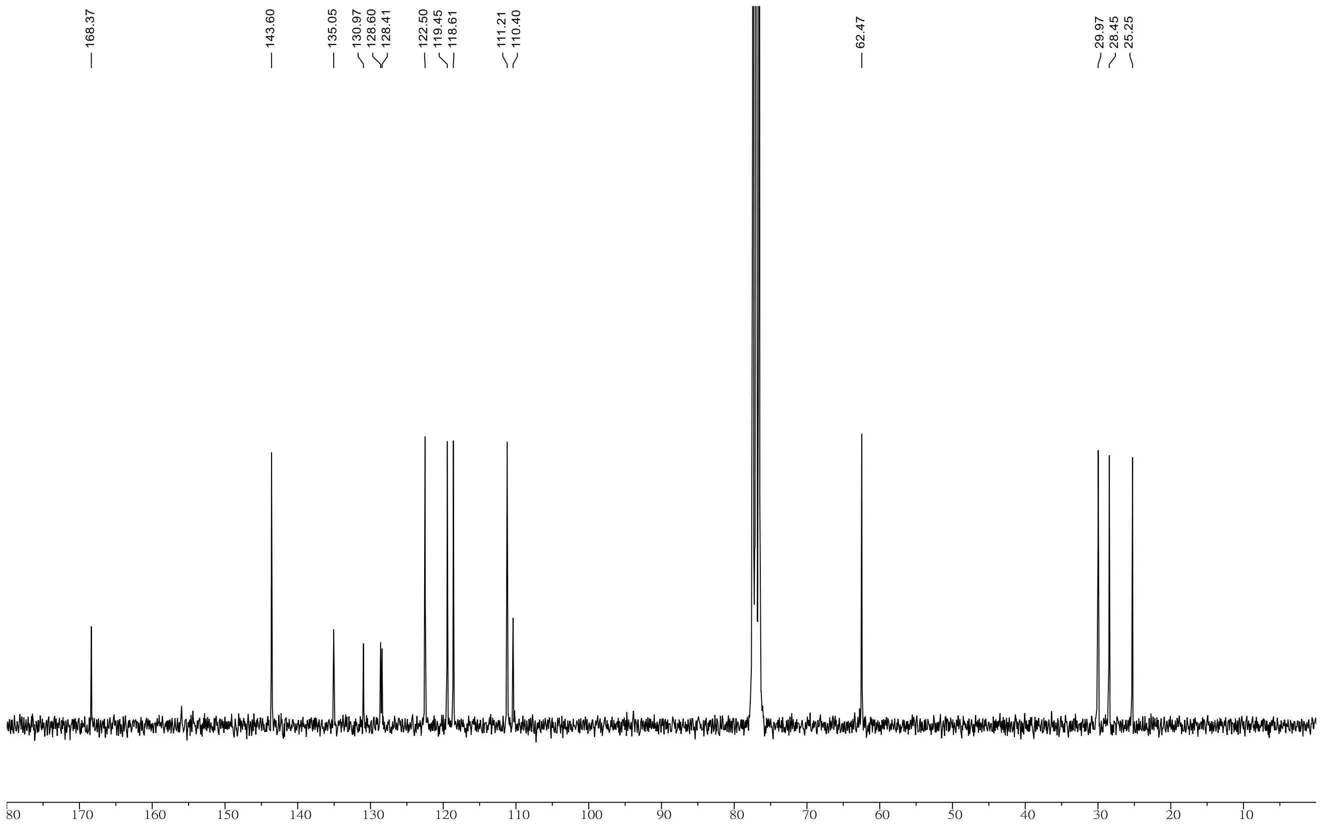

2-[3-(2-Hydroxyethyl)-1*H*-indol-2-yl]cyclopent-2-en-1-one (13)

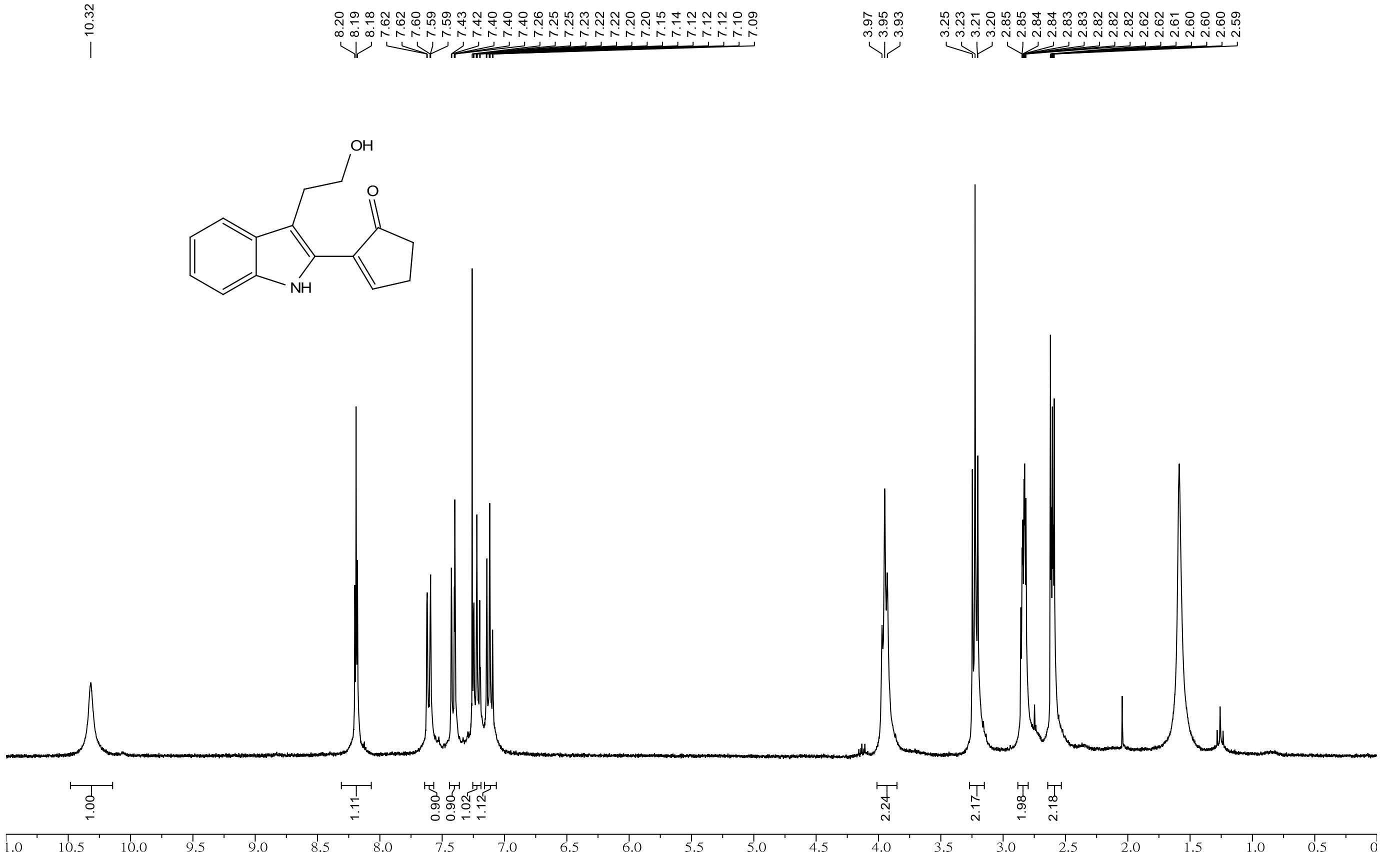

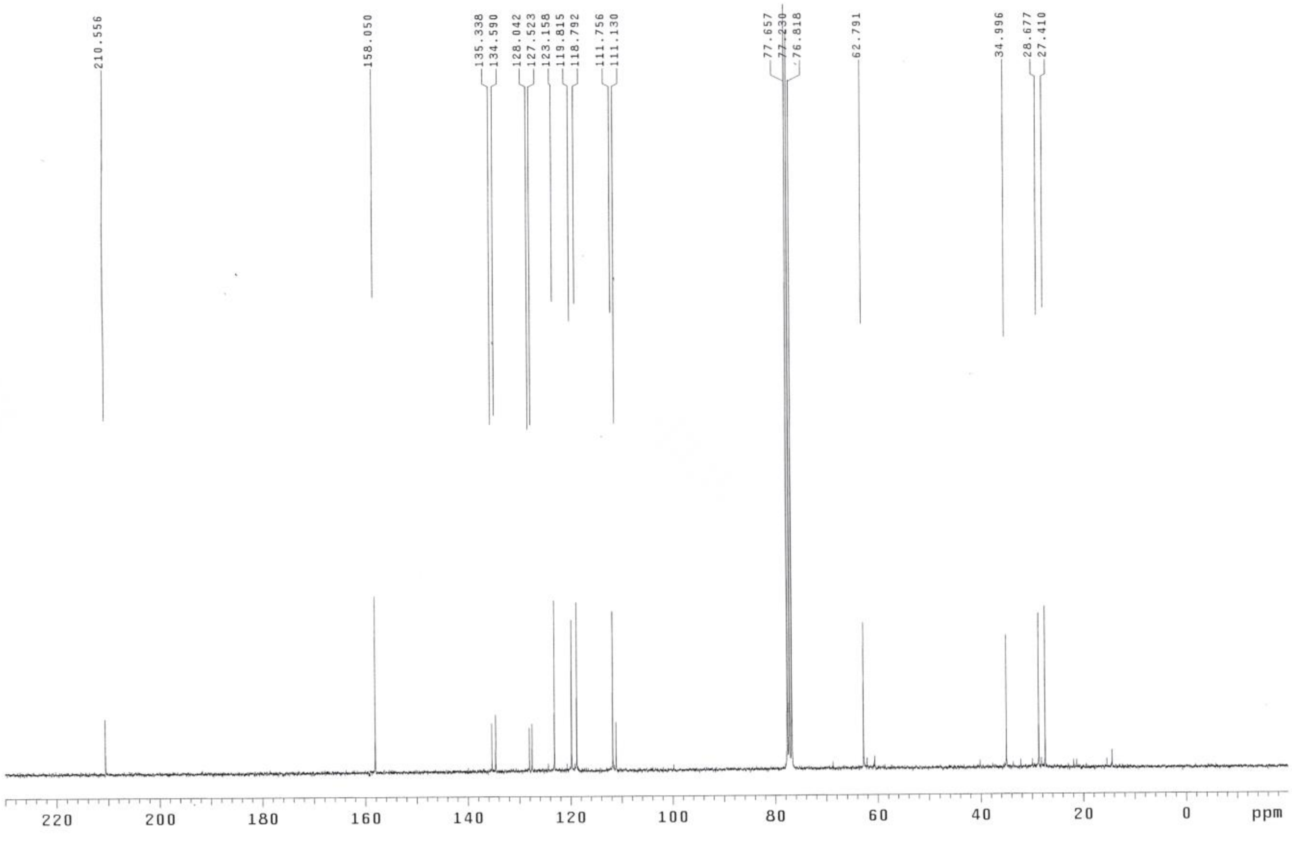

(*E*)-2-[2-(5-{[(Methylsulfonyl)oxy]imino}cyclopent-1-en-1-yl)-1*H*-indol-3-yl]ethyl Methanesulfonate (11)

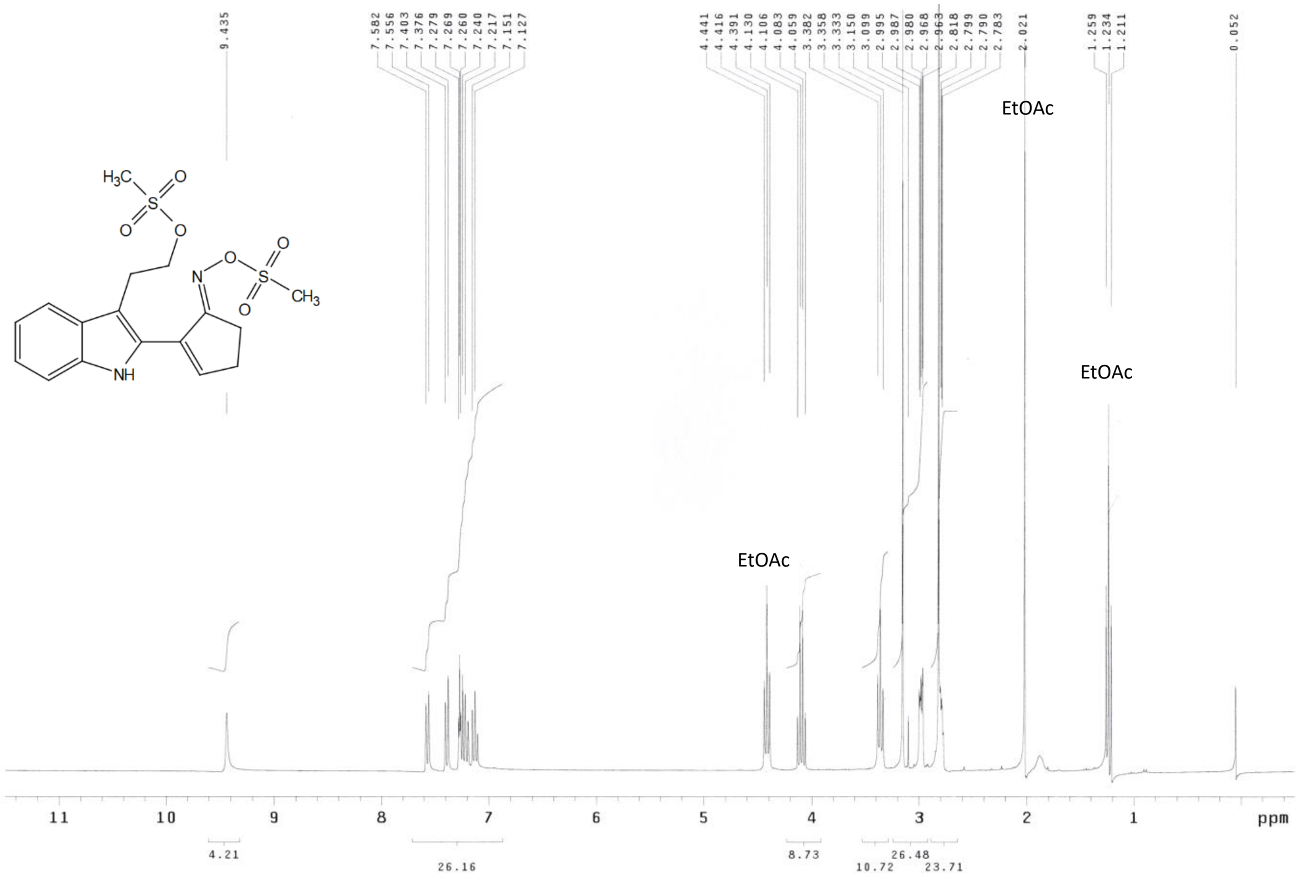

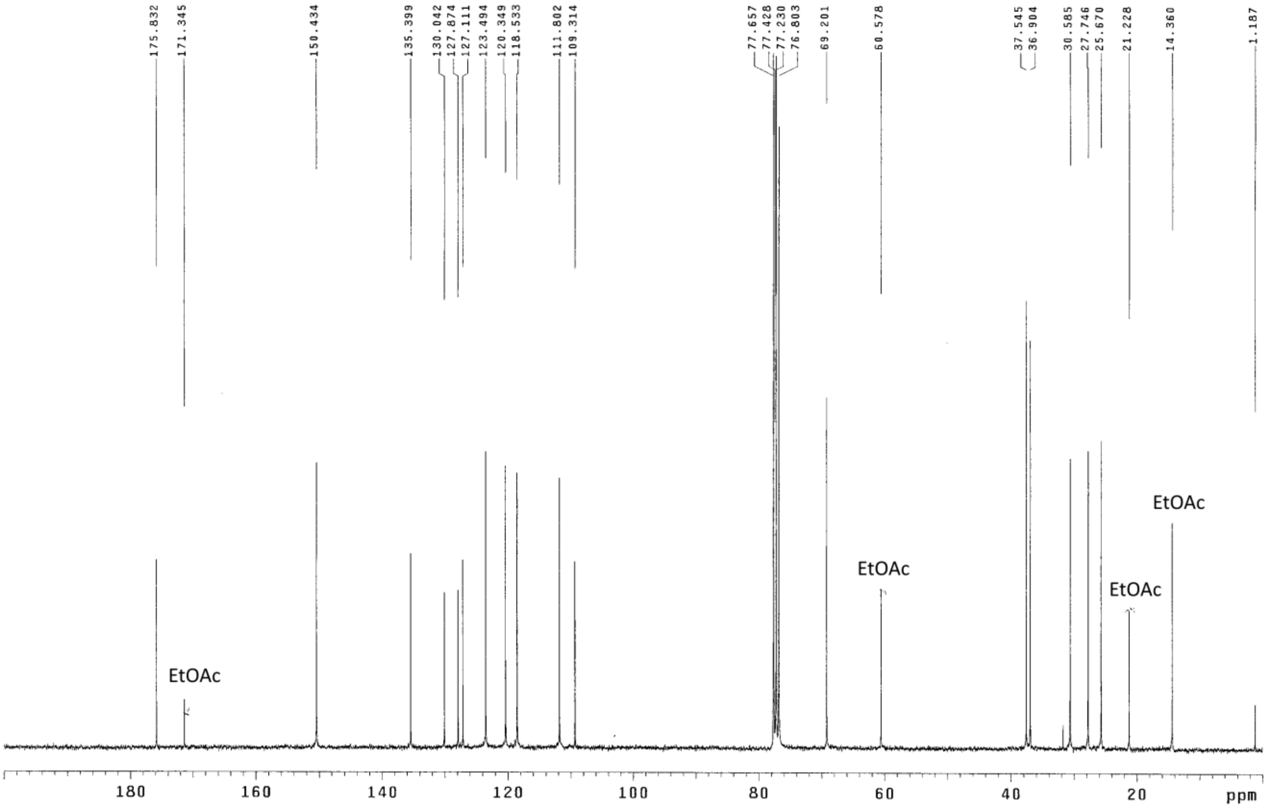

1-Ethoxy-2,3-dihydro-1*H*-spiro[cyclopenta[3,4]pyrazolo[1,5-*a*]indole-10,1'-cyclopropane] (6a)

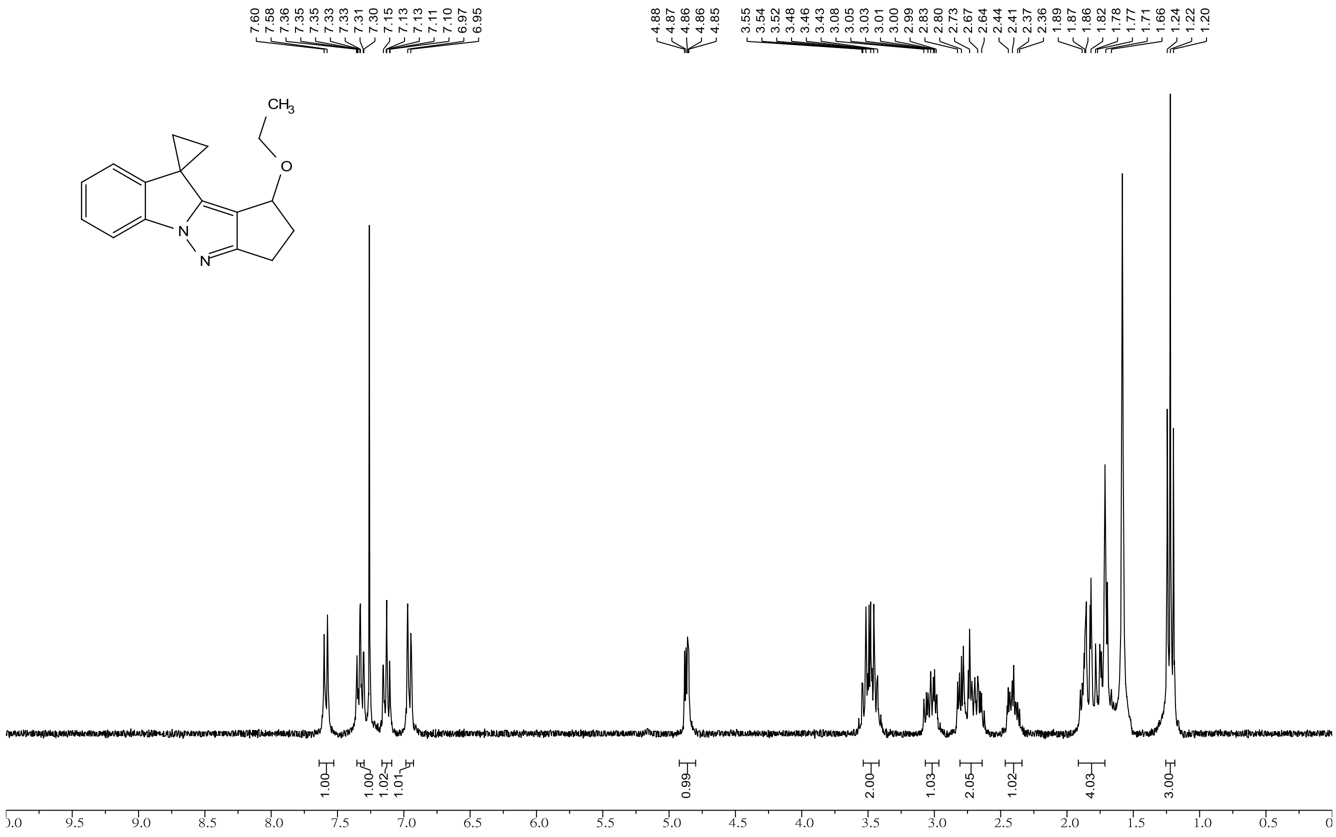

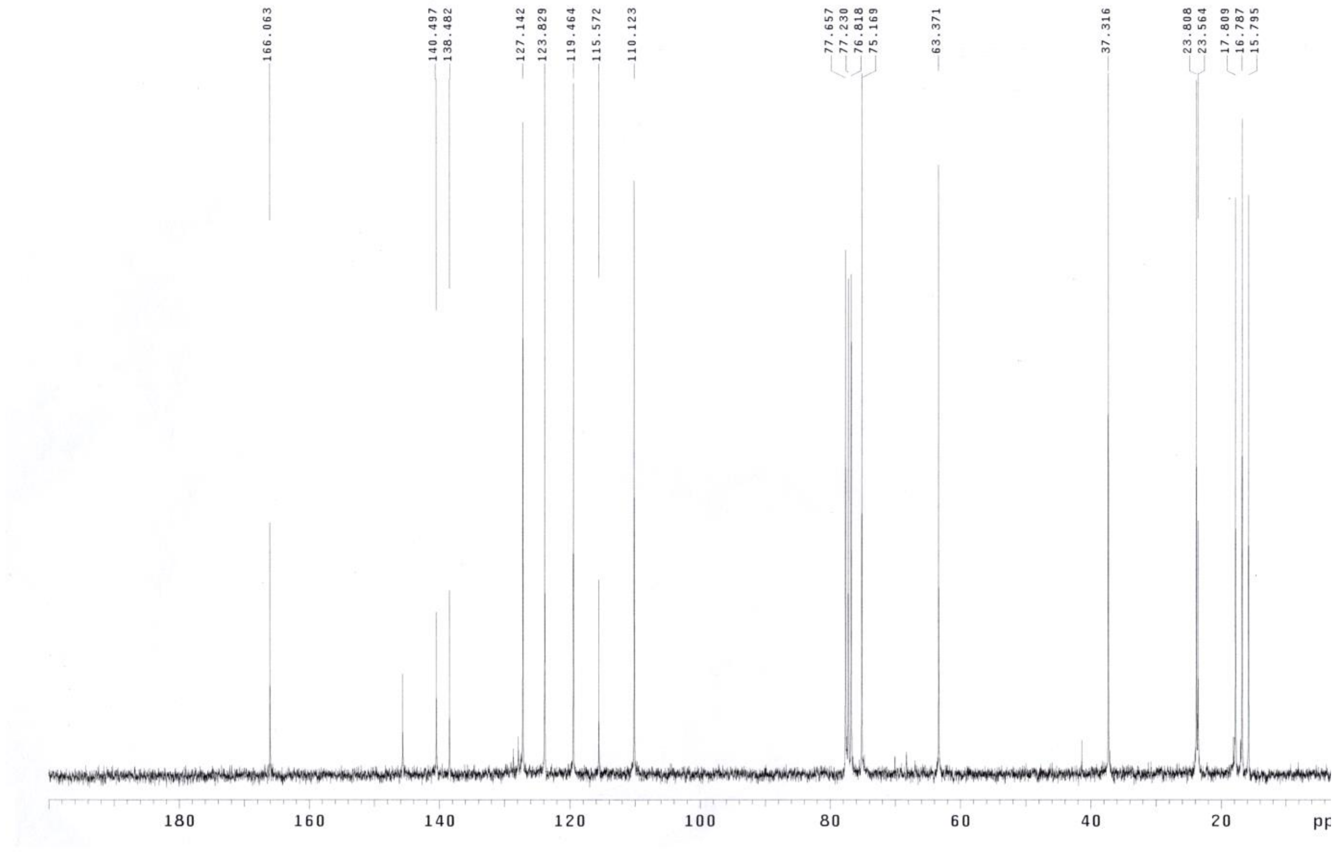

1-Propoxy-2,3-dihydro-1*H*-spiro[cyclopenta[3,4]pyrazolo[1,5-*a*]indole-10,1'-cyclopropane] (6b)

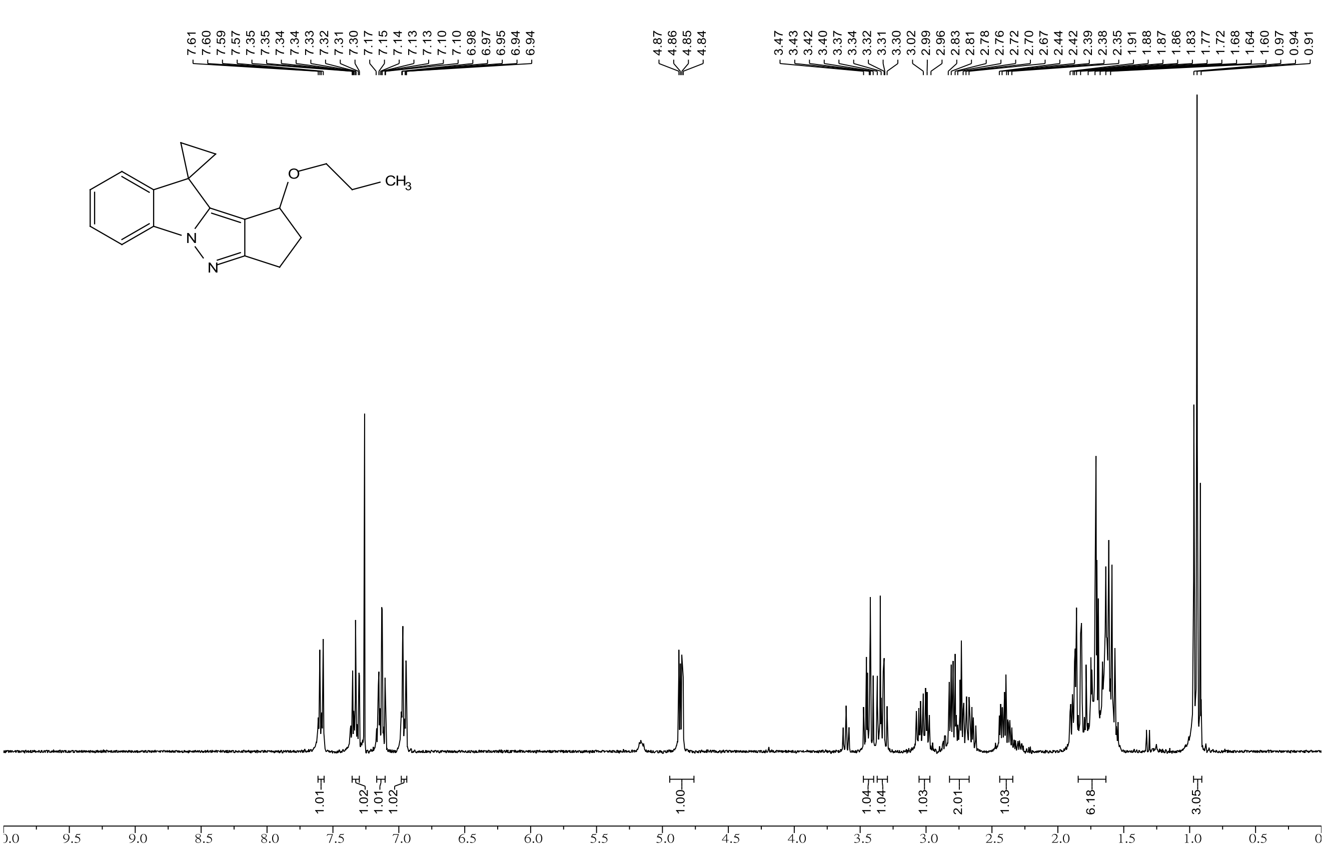

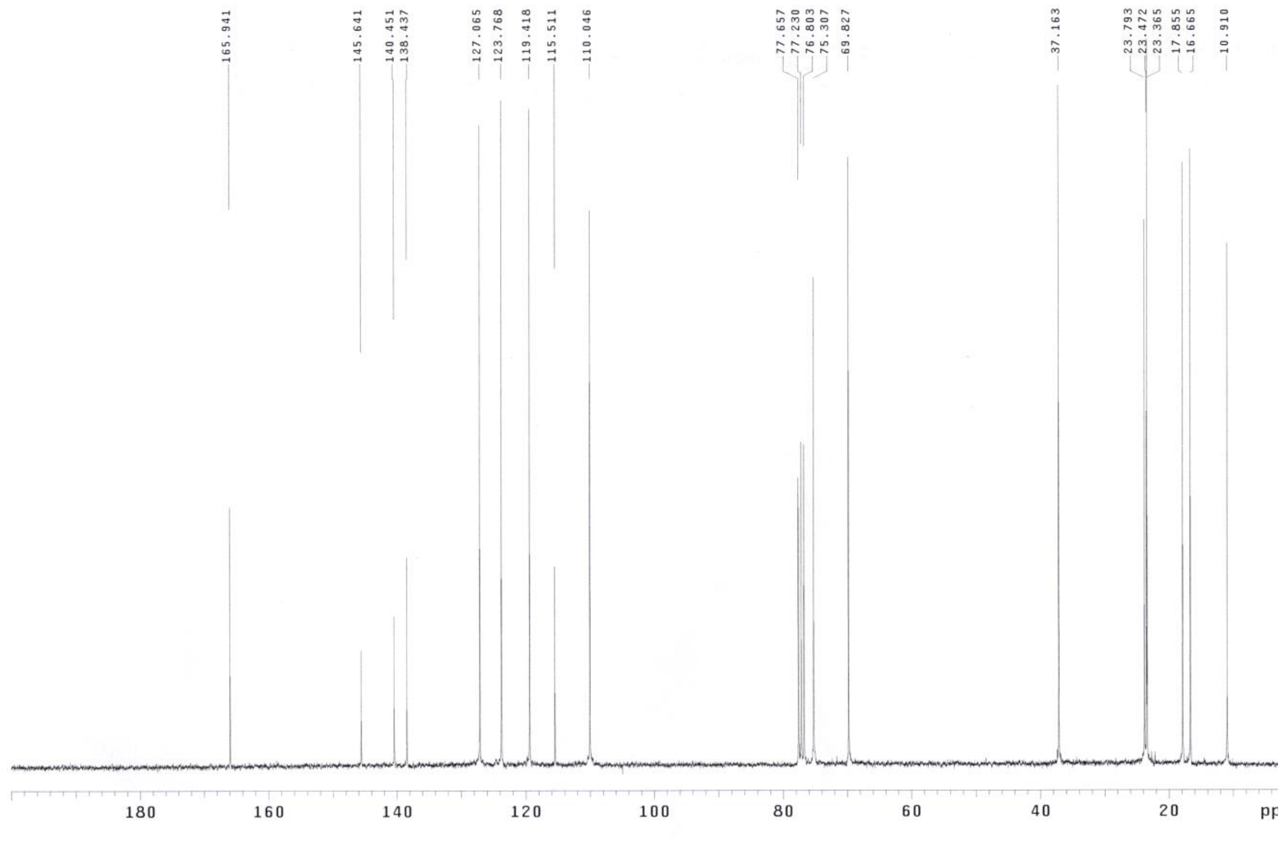

1-(*Tert*-butoxy)-2,3-dihydro-1*H*-spiro[cyclopenta[3,4]pyrazolo[1,5-*a*]indole-10,1'-cyclopropane] (6c)

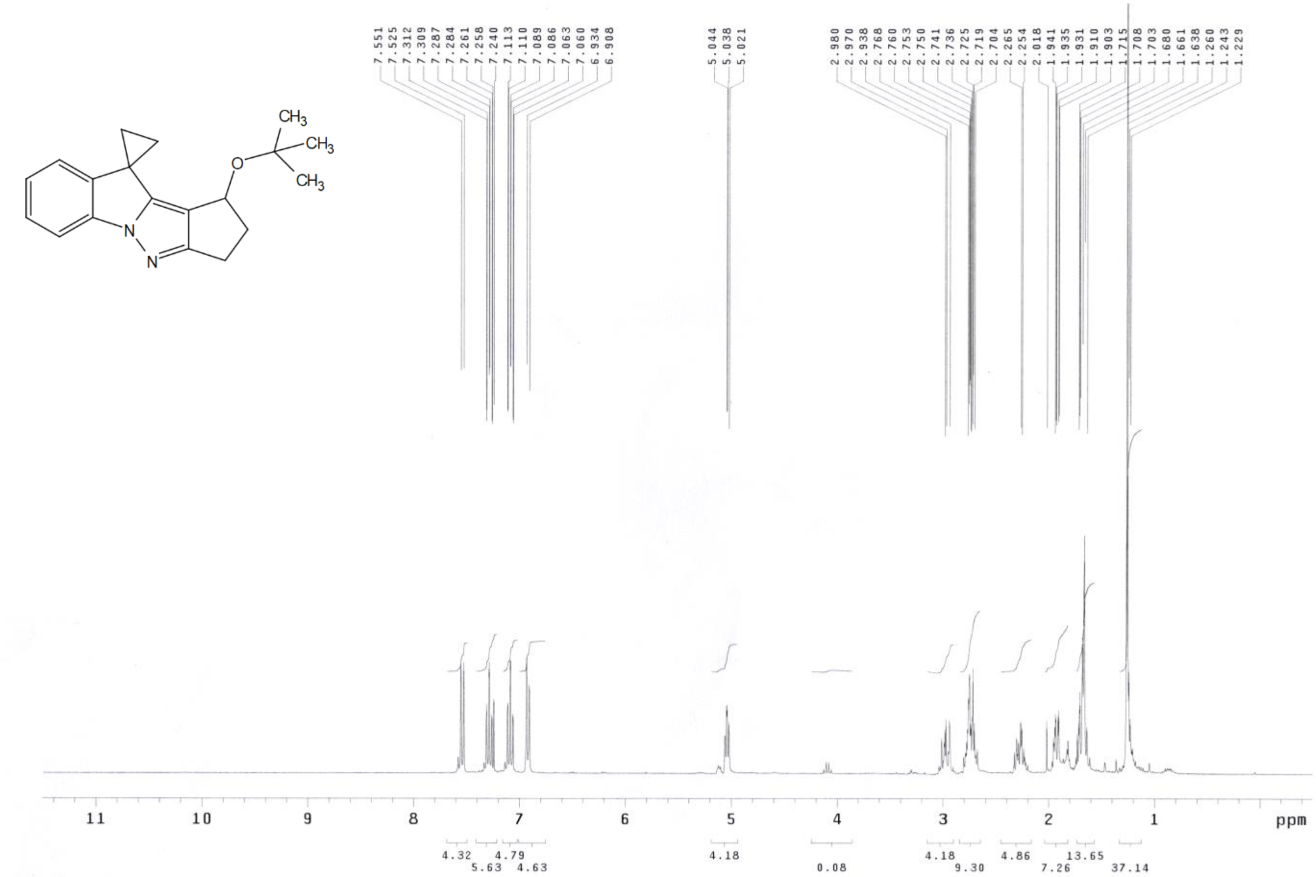

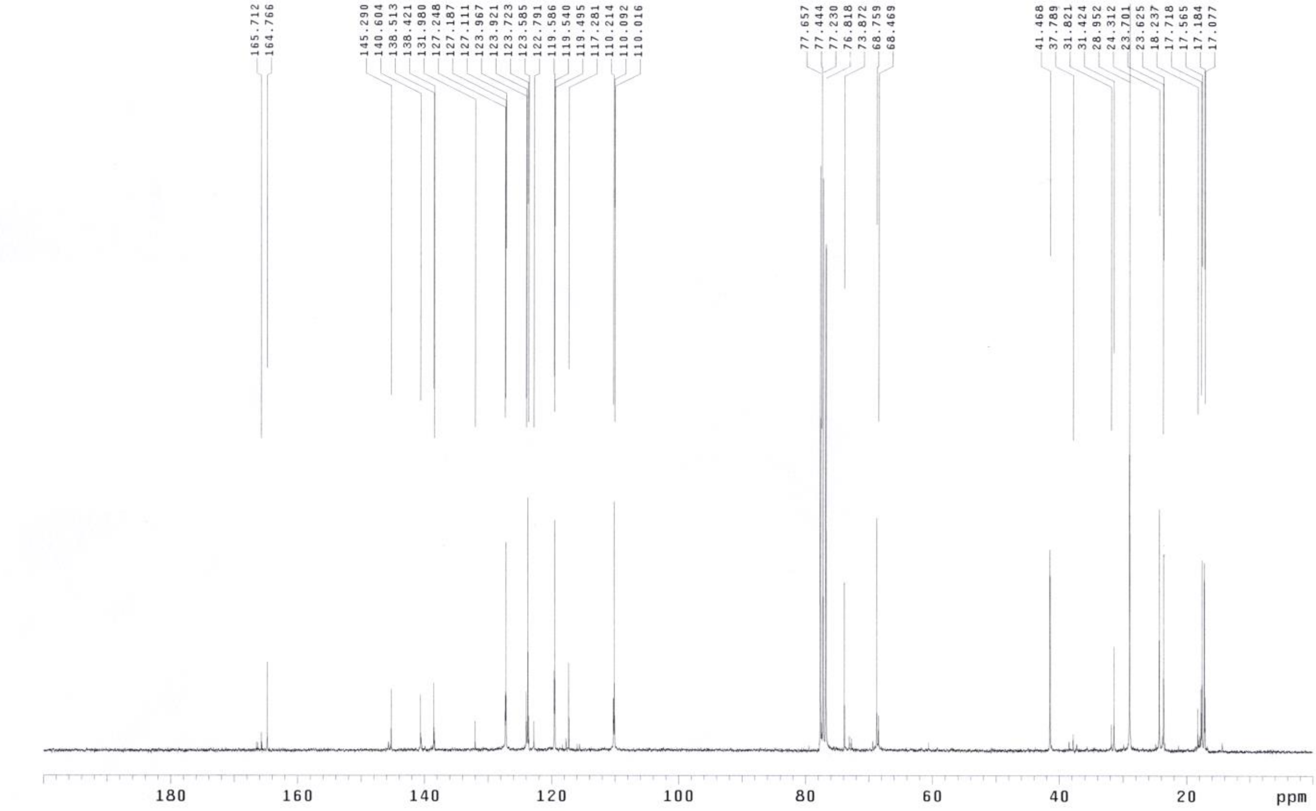

1-(Benzyloxy)-2,3-dihydro-1*H*-spiro[cyclopenta[3,4]pyrazolo[1,5-a]indole-10,1'-cyclopropane] (6d)

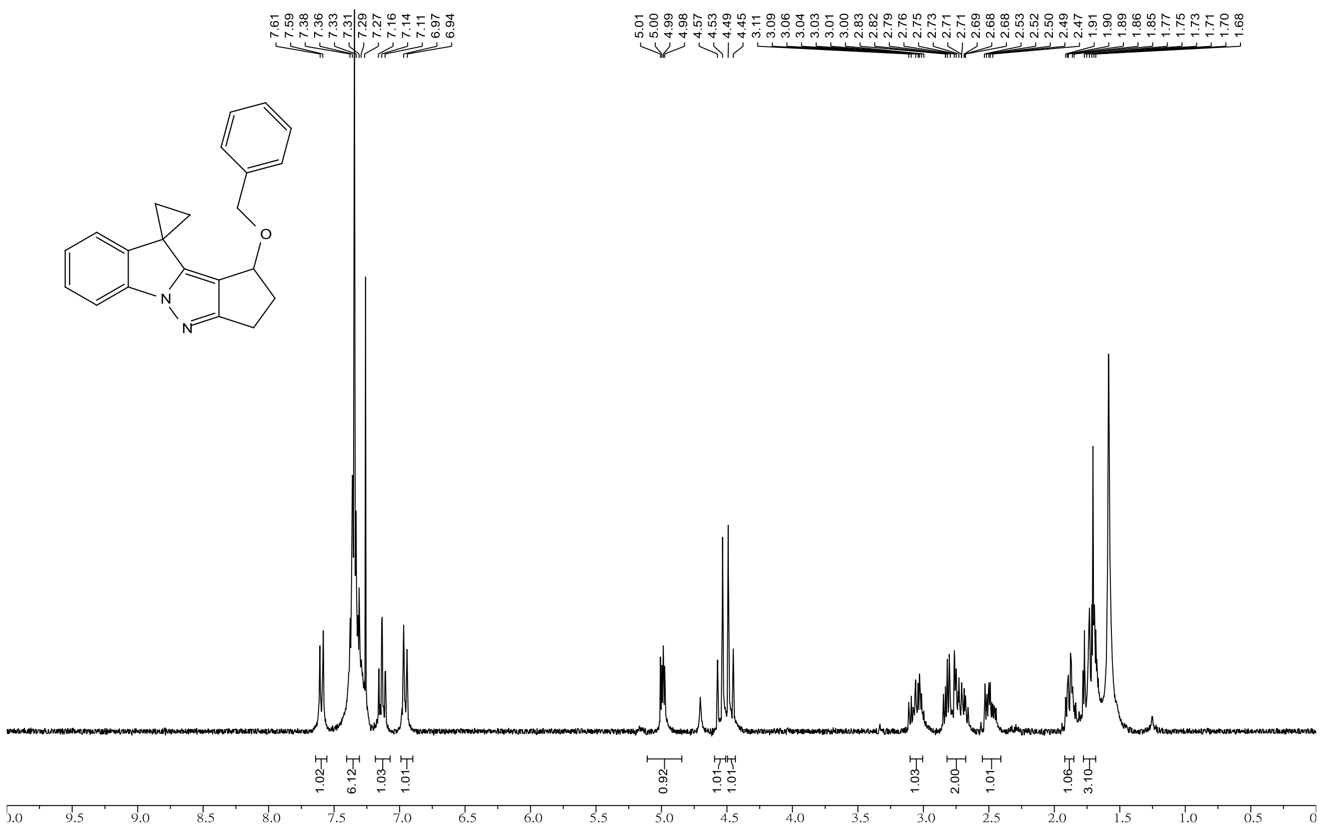

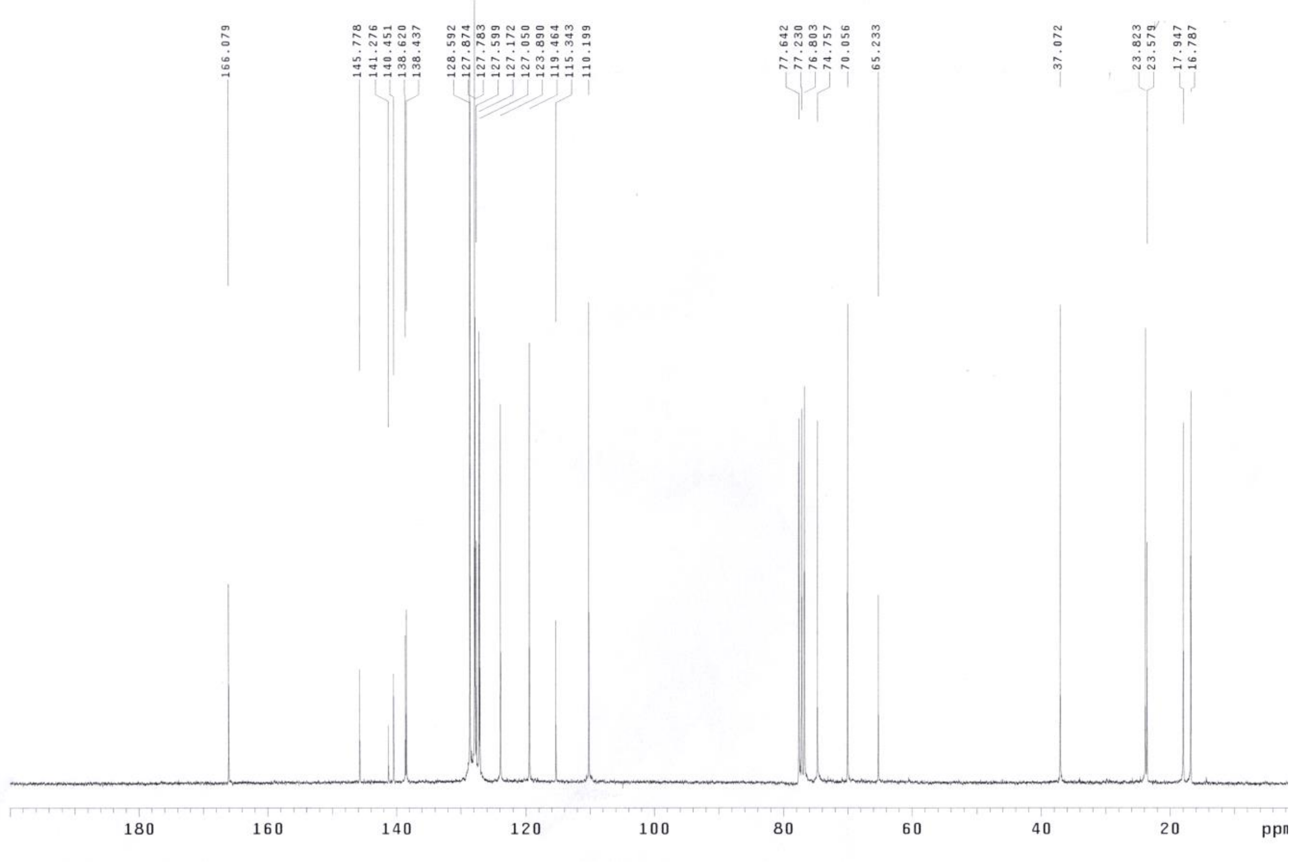

(*E*)-1-(But-2-en-1-yloxy)-2,3-dihydro-1*H*-spiro[cyclopenta[3,4]pyrazolo[1,5-*a*]indole-10,1'-cyclopropane] (6e)

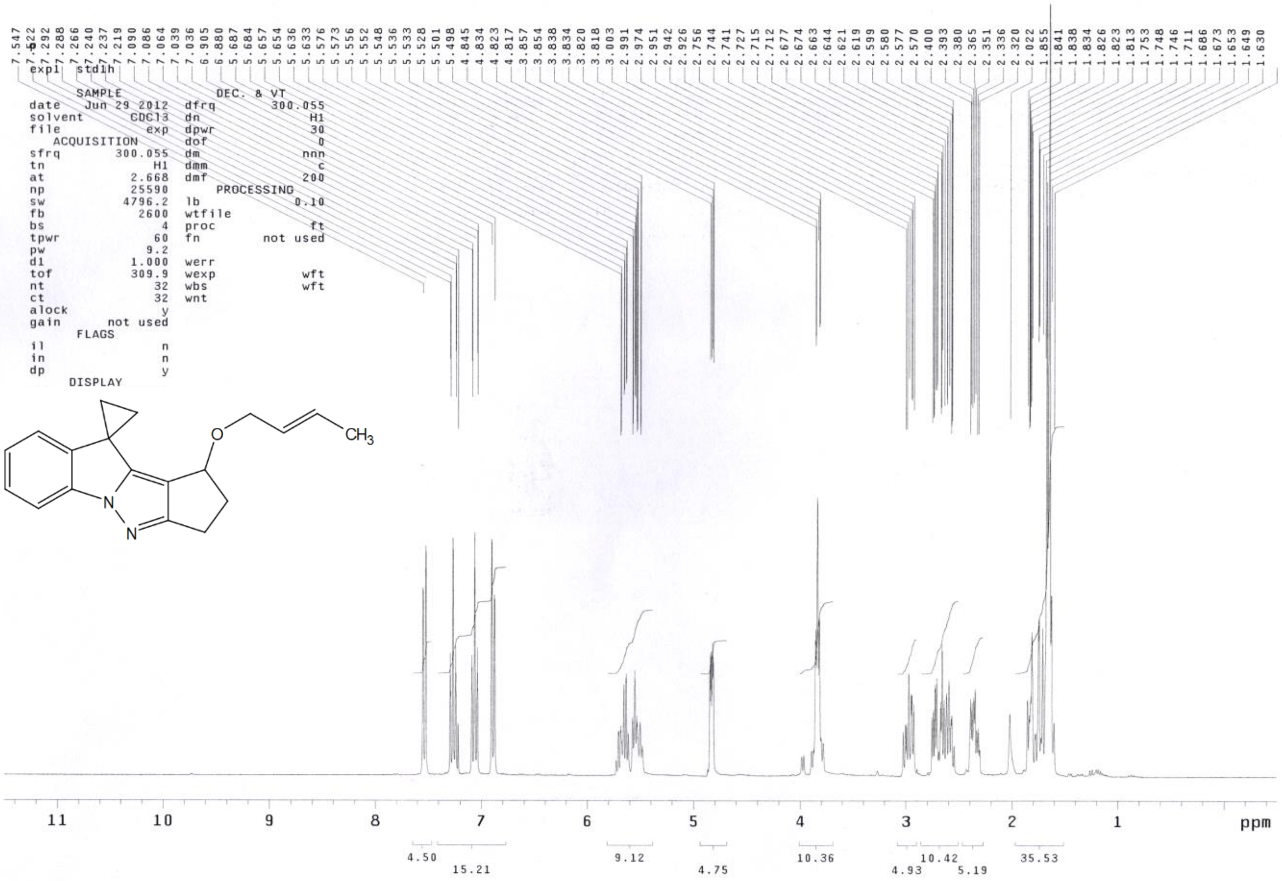

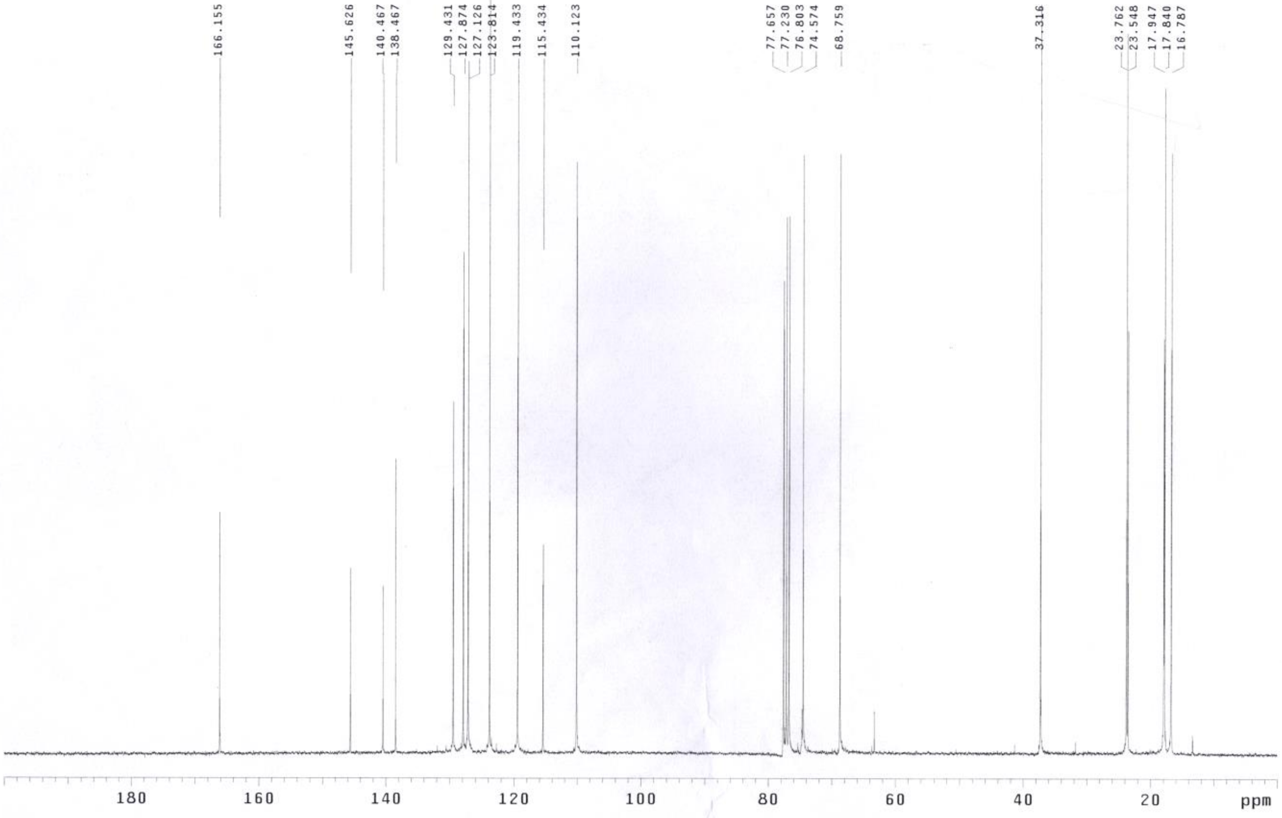

Supplement: Supplementary file 1 [file molecules-28-06374-s001.zip › molecules-2576525-supplementary.pdf]
